# Long-term body mass index variability and the risk of cardiometabolic multimorbidity in middle-aged and older adults: Insights from two prospective cohorts

**DOI:** 10.1016/j.ijcrp.2025.200548

**Published:** 2025-11-17

**Authors:** Xiaoying Ren, Mengge Yang, Juan Tian, Xiaona Chang, Guang Wang, Jia Liu

**Affiliations:** Department of Endocrinology, Beijing Chao-Yang Hospital, Capital Medical University, Beijing 100020, China

**Keywords:** Body mass index (BMI), Variability, Cardiometabolic multimorbidity (CMM)

## Abstract

**Background:**

Body mass index (BMI) variability is considered to be associated with an increased risk of various diseases. However, the association between long-term BMI variability and cardiometabolic multimorbidity (CMM) remains elusive, especially in middle-aged and older adults. This study aimed to explore their relationship in two prospective cohorts.

**Methods:**

Data were analyzed from the UK Biobank and the China Health and Retirement Longitudinal Study (CHARLS). CMM was defined as the coexistence of two or three cardiometabolic diseases, including diabetes mellitus, coronary heart disease, and stroke. BMI measurements from three visits were utilized to assess BMI variability. Cox regression analysis was employed to estimate the relationship between BMI variability and CMM.

**Results:**

The incidence of CMM increased across increasing tertiles of BMI variability, particularly in overweight and obese individuals (all *P* for trend <0.05). This trend was absent in lean subgroups. In the UK Biobank, among participants who were overweight or obese at baseline (BMI ≥25 kg/m^2^), those in the highest tertile of BMI variability exhibited a higher burden of CMM compared to those in the lowest tertile (HR = 2.97, 95 %CI 1.98–4.48, *P* < 0.001). A similar association was observed in CHARLS among individuals overweight or obese at baseline (BMI ≥24 kg/m^2^) (HR = 1.56, 95 %CI 1.09–2.35, *P* = 0.017). No significant association was found between BMI variability and CMM risk in participants with normal baseline BMI.

**Conclusions:**

Higher BMI variability was significantly associated with an elevated risk of CMM in individuals with pre-existing overweight or obesity.

## Graphical abstract

**Figure:**
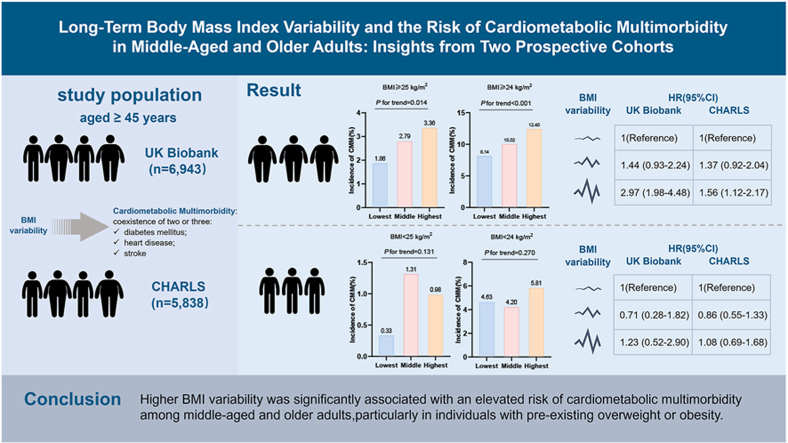

## 1 Introduction

Cardiometabolic multimorbidity (CMM) is defined as the co-occurrence of at least two cardiometabolic diseases, including coronary heart disease, stroke and diabetes mellitus [1,2]. It presents a serious global concern due to the aging population and the rising incidence of cardiometabolic diseases [3]. CMM has been associated with a significant decrease in life expectancy and an exponential rise in mortality risk compared to the incidence of a single cardiometabolic disease [4]. Consequently, it is essential to identify the risk factors for CMM and implement early preventive strategies into clinical practice.

Obesity has been proven to increase the risk of cardiovascular and metabolic diseases [[5], [6], [7], [8], [9]]. While lifestyle interventions are frequently implemented to achieve weight reduction, sustained weight maintenance remains a considerable clinical challenge, often resulting in weight regain [[10], [11], [12], [13]]. Recent long-term studies have indicated that weight regain also occurs after bariatric surgery and stopping pharmacotherapy such as Glucagon-Like Peptide-1 receptor agonist semaglutide [[13], [14], [15]]. Thus, weight regain occurs regardless of how weight loss is achieved, leading to fluctuations in body weight. The repeated cycles of weight loss and weight regain is often referred to as weight “cycling”, “fluctuation”, or “variability” [16]. Of greater concern, a growing body of emerging research highlights that such variability in weight or body mass index (BMI) may, in fact, represent a considerably more serious and insidious threat to health than stable obesity itself [[17], [18], [19]]. This significant and potentially underestimated risk associated with BMI variability demands further investigation and urgent consideration in clinical practice.

It is unclear whether BMI variability is associated with the risk of CMM, and if baseline BMI modifies this association, particularly in middle-aged and older populations. Therefore, we aimed to investigate the association between long-term BMI variability and CMM risk using data from two large prospective cohorts: the UK Biobank and the China Health and Retirement Longitudinal Study (CHARLS).

## 2 Methods

### 2.1 Study design and population

Data from two prospective cohorts, the UK Biobank and the CHARLS, were analyzed in this study. The UK Biobank enrolled over 500,000 adults aged 40–69 years from 22 assessment centers across England, Scotland and Wales, with baseline assessments conducted between 2006 and 2010. Similarly, the CHARLS employed multistage probability sampling to establish a cohort of Chinese adults aged 45 years or older. The baseline survey of CHARLS conducted between 2011 and 2012 recruited 17,708 participants from 10,257 households, representing 450 communities across 28 provinces. The UK Biobank (Application No. 425612) and CHARLS were approved by the North West Multi-Center Research Ethics Committee (MREC) and Ethics Review Committees of Peking University, respectively. Each participant provided the informed consent in both cohorts. For the UK Biobank, data from the baseline (2006–2010) to the final follow-up date (October 31, 2024) were used. In the CHARLS, data from wave 1 (2011–2012), wave 2 (2013), wave 3 (2015), and wave 4 (2018) were analyzed, with wave 1 serving as the baseline.

The selection process of the study population was shown in Fig. S1. Initially, 450,404 participants aged 45 years or older were recruited from the UK Biobank, and 17,301 participants of the same age group were recruited from CHARLS. Subsequently, individuals who met the following criteria were excluded: (1) missing baseline or follow-up data on BMI, (2) missing baseline or follow-up data on CMM or presence of CMM at baseline, and (3) presence of cancer at baseline. Ultimately, a total of 12,781 participants were deemed eligible for subsequent analysis, with 6943 from the UK Biobank and 5838 from the CHARLS.

### 2.2 Definition of body mass index variability

BMI variability was defined as the fluctuation in BMI across visits. In our study, BMI measurements from three visits were utilized to assess BMI variability among participants. In the CHARLS cohort, the three visits were conducted in wave 1 (2011–2012), wave 2 (2013), and wave 3 (2015). In the UK Biobank cohort, the three visits took place in wave 1 (2006–2010), wave 2 (2012–2013), and wave 3 (2014–2019). Consistent with previous studies [17,19], the following indicators were calculated to represent BMI variability: standard deviation (SD), coefficient of variation (CV), variability independent of the mean (VIM), and average real variability (ARV). VIM was calculated as the SD divided by the mean to the power x. Power x was modeled as SD = k × mean^x^ and derived from fitting curves by nonlinear regression analysis implemented in the R studio. ARV was determined by calculating the average of the absolute differences between successive BMI measurements across the multiple visits. The formulas for all BMI variability metrics are presented in **Appendix**.

### 2.3 Definition of cardiometabolic multimorbidity

The primary outcome of this study was CMM. CMM was defined as the coexistence of two or three cardiometabolic diseases, including diabetes mellitus, coronary heart disease, and stroke [[20], [21], [22]]. In the UK Biobank, the definitions of diabetes mellitus, coronary heart disease, and stroke were based on ICD9, ICD10, and self-reported medical history, with the specific Field IDs listed in Table S1. In the CHARLS, participants were considered to have diabetes if any of the following criteria were met: fasting blood glucose ≥126 mg/dL, random blood glucose ≥200 mg/dL, glycosylated hemoglobin A1c (HbA1c) ≥ 6.5 %, self-reported diagnosis, or use of glucose-lowering medication. Heart disease and stroke in the CHARLS were identified primarily through self-report. In the UK Biobank and CHARLS, the diagnosis date of the second cardiovascular metabolic disease was considered the time of CMM occurrence. The follow-up time for participants was calculated from the date of enrollment to the date of outcome occurrence or the last known follow-up date, whichever occurred first.

### 2.4 Covariates

The covariates included age, gender, education level, marital status, smoking status, drinking status, waist circumference, estimated glomerular filtration rate (eGFR), total cholesterol (TC), triglyceride (TG), high-density lipoprotein cholesterol (HDL-C), low-density lipoprotein cholesterol (LDL-C), systolic blood pressure (SBP), diastolic blood pressure (DBP), lipid-lowering medications, antihypertensive medications, mean BMI and baseline BMI. Education level was divided into three levels: below high school, high school, and college or above. Marital status was classified into two categories: married or partnered and other marital status (separated, divorced, unmarried, or widowed). Smoking status was categorized as current smokers and non-current smokers (never or former smokers). Drinking status was categorized as current drinkers and non-current drinkers (never or former drinkers). BMI was calculated as the weight (kg) divided by the square of height (m^2^).The mean BMI was calculated using the BMI measurements from three visits.

### 2.5 Statistical analysis

The participants were stratified into three groups according to the BMI variability tertiles in the UK Biobank and CHARLS. Baseline characteristics were subsequently analyzed across these tertile groups. Continuous variables were presented as mean ± SD or median (range), depending on the normality of their distribution as assessed by Shapiro-Wilk tests. Categorical variables were presented as frequencies (percentages). Inter-group differences were assessed using One-way ANOVA for normally distributed continuous variables, the Kruskal-Wallis test for non-normally distributed continuous variables, and chi-squared tests for categorical variables.

To account for the potential influence of baseline BMI on the relationship between BMI variability and incident CMM, the total population was stratified into subgroups based on baseline BMI. Specifically, within the UK Biobank, participants were categorized into two groups: those with baseline BMI <25 kg/m^2^ and those with baseline BMI ≥25 kg/m^2^. In the CHARLS dataset, the corresponding BMI cut-off was <24 kg/m^2^and ≥24 kg/m^2^. Multivariate Cox regression models were employed to calculate hazard ratios (HRs) and 95 % confidence intervals (CIs) for incident CMM associated with BMI variability tertiles within each BMI subgroup. The crude model was unadjusted for covariates, model 1 was adjusted for age, gender, education level and marital status. model 2 was adjusted for age, gender, education level, marital status, smoking status, drinking status, waist circumference, eGFR, TC, TG, HDL-C, LDL-C, SBP, DBP, lipid-lowering medications, antihypertensive medications, mean BMI and baseline BMI.

All statistical analyses were conducted using R software (version 4.4.1). A two-tailed *P* of <0.05 was considered as statistically significant.

## 3 Results

### 3.1 Baseline characteristics of participants by tertiles of BMI variability

Based on the inclusion and exclusion criteria, 6943 participants from the UK Biobank (mean age: 56.68 years, female: 49.89 %) and 5838 participants from the CHARLS (mean age: 58.61 years, female: 54.41 %) were finally included in the study. Baseline characteristics of participants, stratified by tertiles of BMI variability, were shown in Table 1 for the UK Biobank cohort and in Table 2 for the CHARLS cohort.

**Table 1:** Baseline characteristics of participants by tertiles of BMI variability in the UK Biobank cohort.

Variable	Overall (n = 6943)	Tertiles of BMI variability			*P* value
		Lowest (n = 2343)	Middle (n = 2296)	Highest (n = 2304)	
**Age (years)**	56.68 (6.16)	57.19 ± 6.17	56.86 ± 6.14	55.99 ± 6.11	**<0.001**
**Gender, n (%)**					**<0.001**
Female	3464 (49.89)	986 (42.94)	1102 (47.83)	1376 (58.73)	
Male	3479 (50.11)	1310 (57.06)	1202 (52.17)	967 (41.27)	
**Marital status, n (%)**					**<0.001**
Married or partnered	1616 (23.28)	486 (21.17)	489 (21.22)	641 (27.36)	
Other marital status	5327 (76.72)	1810 (78.83)	1815 (78.78)	1702 (72.64)	
**Education level, n (%)**					0.050
College	3281 (47.26)	1128 (49.13)	1099 (47.70)	1054 (44.99)	
Other levels	3080 (44.36)	987 (42.99)	1002 (43.49)	1091 (46.56)	
Unknown	582 (8.38)	181 (7.88)	203 (8.81)	198 (8.45)	
**Smoking status, n (%)**					**<0.001**
Non-current	6559 (94.63)	2188 (95.38)	2194 (95.43)	2177 (93.11)	
Current	372 (5.37)	106 (4.62)	105 (4.57)	161 (6.89)	
**Drinking status, n (%)**					**0.014**
Non-current	337 (4.86)	95 (4.14)	104 (4.52)	138 (5.89)	
Current	6603 (95.14)	2201 (95.86)	2199 (95.48)	2203 (94.11)	
**Antihypertensive medications, n (%)**					0.894
No	5978 (86.10)	1979 (86.19)	1988 (86.28)	2011 (85.83)	
Yes	965 (13.90)	317 (13.81)	316 (13.72)	332 (14.17)	
**Lipid-lowering medications, n (%)**					0.378
No	5999 (86.40)	1994 (86.85)	1972 (85.59)	2033 (86.77)	
Yes	944 (13.60)	302 (13.15)	332 (14.41)	310 (13.23)	
**SBP, mmHg**	81.69 (9.91)	81.57 ± 10.02	81.56 ± 9.78	81.92 ± 9.92	0.373
**DBP, mmHg**	136.82 (18.04)	137.51 ± 18.18	136.80 ± 17.92	136.16 ± 18.01	**0.044**
**HbA1c, %**	35.27 (4.86)	35.03 ± 4.40	35.20 ± 4.60	35.58 ± 5.49	**<0.001**
**FPG, mmol/L**	4.96 (0.93)	4.94 ± 0.75	4.94 ± 0.88	5.01 ± 1.11	**0.028**
**TC, mmol/L**	5.72 (1.10)	5.76 ± 1.06	5.71 ± 1.09	5.69 ± 1.14	0.097
**TG, mmol/L**	1.67 (0.97)	1.67 ± 0.92	1.65 ± 0.97	1.70 ± 1.02	0.147
**LDL-C, mmol/L**	3.57 (0.84)	3.60 ± 0.81	3.56 ± 0.83	3.55 ± 0.86	0.213
**HDL-C, mmol/L**	1.47 (0.38)	1.48 ± 0.38	1.48 ± 0.37	1.45 ± 0.37	**0.043**
**eGFR, ml/(min × 1.73m^2^)**	95.18 (11.41)	94.51 ± 11.52	95.00 ± 11.15	96.03 ± 11.51	**<0.001**
**Waist circumference, cm**	87.99 (12.71)	87.47 ± 12.21	89.38 ± 14.06	87.08 ± 11.61	**<0.001**
**Baseline BMI, kg/m^2^**	26.56 (4.24)	26.25 ± 3.80	27.55 ± 5.04	25.87 ± 3.52	**<0.001**
**mean BMI, kg/m**^**2**^	26.52 (4.20)	25.88 ± 3.52	26.24 ± 3.83	27.42 ± 4.96	**<0.001**
**BMI SD**	0.97 (0.80)	0.35 ± 0.15	0.79 ± 0.20	1.75 ± 0.90	**<0.001**
**BMI CV**	3.58 (2.61)	1.37 ± 0.55	3.02 ± 0.66	6.31 ± 2.62	**<0.001**
**BMI VIM**	0.96 (0.72)	0.37 ± 0.17	0.82 ± 0.26	1.67 ± 0.77	**<0.001**
**BMI ARV**	1.13 (0.92)	0.44 ± 0.22	0.96 ± 0.33	1.98 ± 1.08	**<0.001**

Abbreviation: SBP, systolic blood pressure; DBP, diastolic blood pressure; HbA1c, glycosylated hemoglobin; FPG, fasting plasma glucose; TC, total cholesterol; TG, triglycerides; LDL-C, low-density lipoprotein cholesterol; HDL-C, high-density lipoprotein cholesterol; eGFR, estimated glomerular filtration rate; BMI,body mass index; SD,standard deviation; CV,coefficient of variation; VIM,variability independent of the mean; ARV,average real variability.

Continuous variables were presented as mean ± SD or median (upper and lower quartiles), and categorical variables were presented as number (%).

**Table 2:** Baseline characteristics of participants by tertiles of BMI variability in the CHARLS cohort.

Variable	Overall (n = 5838)	Tertiles of BMI variability			*P* value
		Lowest (n = 1948)	Middle (n = 1944)	Highest (n = 1946)	
**Age (years)**	58.61 ± 8.61	59.02 ± 8.52	58.34 ± 8.42	58.48 ± 8.89	**0.030**
**Gender, n (%)**					**<0.001**
Female	3173 (54.41)	982 (50.41)	1031 (53.12)	1160 (59.70)	
Male	2659 (45.59)	966 (49.59)	910 (46.88)	783 (40.30)	
**Marital status, n (%)**					0.230
Married or partnered	612 (10.48)	190 (9.75)	200 (10.29)	222 (11.41)	
Other marital status	5226 (89.52)	1758 (90.25)	1744 (89.71)	1724 (88.59)	
**Education level, n (%)**					0.390
Less than lower secondary education	5287 (90.58)	1767 (90.76)	1746 (89.81)	1774 (91.16)	
Tertiary education	62 (1.06)	25 (1.28)	19 (0.98)	18 (0.92)	
Upper secondary & vocational training	488 (8.36)	155 (7.96)	179 (9.21)	154 (7.91)	
**Smoking status, n (%)**					**<0.001**
Non-current	4069 (69.70)	1290 (66.22)	1369 (70.42)	1410 (72.46)	
Current	1769 (30.30)	658 (33.78)	575 (29.58)	536 (27.54)	
**Drinking status, n (%)**					**<0.001**
Non-current	4437 (76.00)	1429 (73.36)	1464 (75.31)	1544 (79.34)	
Current	1401 (24.00)	519 (26.64)	480 (24.69)	402 (20.66)	
**Antihypertensive medications, n (%)**					0.910
No	5595 (95.84)	1868 (95.89)	1865 (95.94)	1862 (95.68)	
Yes	243(4.16)	80(4.11)	79(4.06)	84(4.32)	
**Lipid-lowering medications, n (%)**					0.050
No	4889 (83.74)	1664 (85.42)	1610 (82.82)	1615 (82.99)	
Yes	949 (16.26)	284 (14.58)	334 (17.18)	331 (17.01)	
**SBP, mmHg**	129.57 ± 20.92	128.38 ± 20.77	129.43 ± 20.72	130.90 ± 21.18	**<0.001**
**DBP, mmHg**	75.48 ± 11.98	74.62 ± 12.09	75.33 ± 11.85	76.50 ± 11.94	**<0.001**
**HbA1c, %**	5.23 ± 0.74	5.19 ± 0.70	5.21 ± 0.69	5.28 ± 0.83	**<0.010**
**FPG, mg/dL**	108.12 ± 32.66	107.30 ± 30.43	107.56 ± 31.58	109.55 ± 35.80	0.110
**TC, mg/dL**	129.57 ± 20.92	128.38 ± 20.77	129.43 ± 20.72	130.90 ± 21.18	**<0.001**
**TG, mg/dL**	130.54 ± 103.67	127.70 ± 104.90	129.01 ± 95.23	135.06 ± 110.36	0.110
**LDL-C, mg/dL**	116.69 ± 34.25	116.55 ± 33.48	115.71 ± 33.67	117.85 ± 35.60	0.220
**HDL-C, mg/dL**	75.48 ± 11.98	74.62 ± 12.09	75.33 ± 11.85	76.50 ± 11.94	**<0.001**
**eGFR, ml/(min × 1.73m^2^)**	96.33 ± 13.43	95.69 ± 13.30	97.01 ± 12.85	96.31 ± 14.11	**0.020**
**Waist circumference, cm**	85.04 ± 9.90	83.55 ± 9.61	85.04 ± 9.79	86.53 ± 10.07	**<0.001**
**Baseline BMI, kg/m**^**2**^	23.41 ± 3.61	22.86 ± 3.39	23.47 ± 3.43	23.91 ± 3.90	**<0.001**
**mean BMI, kg/m**^**2**^	23.60 ± 3.48	22.90 ± 3.39	23.62 ± 3.42	24.28 ± 3.50	**<0.001**
**BMI SD**	0.93 ± 0.89	0.32 ± 0.13	0.73 ± 0.13	1.75 ± 1.13	**<0.001**
**BMI CV**	3.95 ± 3.60	1.44 ± 0.61	3.15 ± 0.71	7.28 ± 4.48	**<0.001**
**BMI VIM**	1.15 ± 1.17	0.40 ± 0.19	0.91 ± 0.27	2.14 ± 1.56	**<0.001**
**BMI ARV**	0.93 ± 0.85	0.34 ± 0.14	0.74 ± 0.16	1.72 ± 1.06	**<0.001**

Abbreviation: SBP, systolic blood pressure; DBP, diastolic blood pressure; HbA1c, glycosylated hemoglobin; FPG, fasting plasma glucose; TC, total cholesterol; TG, triglycerides; LDL-C, low-density lipoprotein cholesterol; HDL-C, high-density lipoprotein cholesterol; eGFR, estimated glomerular filtration rate; BMI,body mass index; SD,standard deviation; CV,coefficient of variation; VIM,variability independent of the mean; ARV,average real variability.

Continuous variables were presented as mean ± SD or median (upper and lower quartiles), and categorical variables were presented as number (%).

In the UK Biobank, participants in the higher BMI variability tertiles displayed elevated levels of HbA1c, FPG, eGFR, and mean BMI, along with decreased DBP and HDL-C (all *P* < 0.05). These individuals were also more likely to be married or partnered and to be current smokers, and less likely to be current drinkers (all *P* < 0.05). Additionally, baseline BMI and waist circumference were highest in the middle BMI variability group and lowest in the highest BMI variability group (all *P* < 0.05). In the CHARLS, participants in the higher BMI variability tertiles exhibited elevated levels of DBP, SBP, waist circumference, HbA1c, baseline BMI, and mean BMI (all *P* < 0.05), and were less likely to be current smokers or drinkers (all *P* < 0.05).

### 3.2 The incidence of CMM in populations with different tertiles of BMI variability

**Fig. 1:**
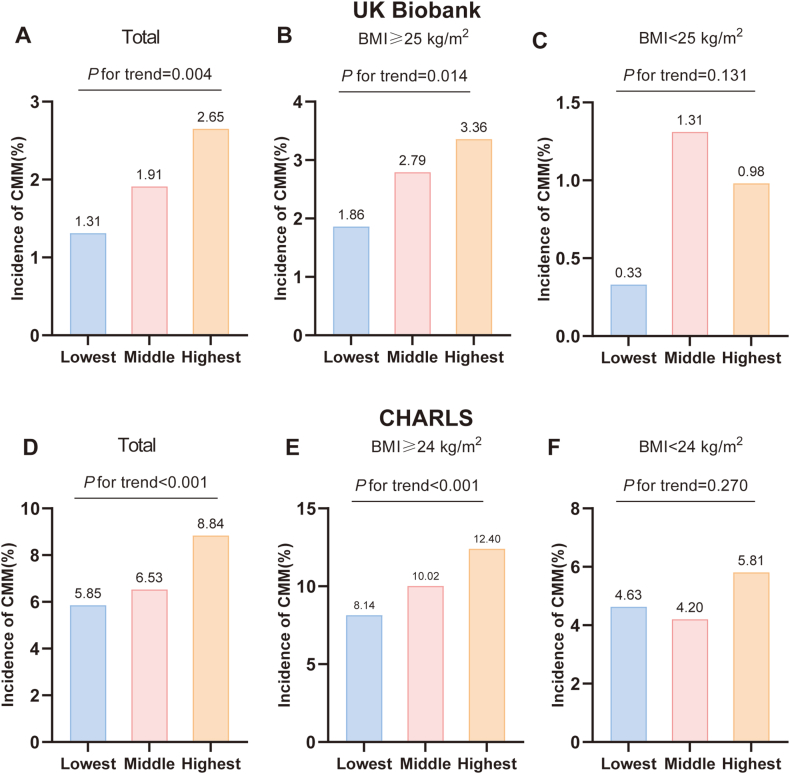
The incidence of CMM in populations with different tertiles of BMI variability. (A) The incidence of CMM across BMI variability tertiles in total participants (UK Biobank). (B) The incidence of CMM across BMI variability tertiles in baseline BMI ≥25 subgroup (UK Biobank). (C) The incidence of CMM across BMI variability tertiles in baseline BMI <25 subgroup (UK Biobank). (D) The incidence of CMM across BMI variability tertiles in total participants (CHARLS). (E) The incidence of CMM across BMI variability tertiles in baseline BMI ≥24 subgroup (CHARLS). (F) The incidence of CMM across BMI variability tertiles in baseline BMI <24 subgroup (CHARLS).

After a median follow-up of 16 years, 136 (1.96 %) participants in the UK Biobank developed CMM. In the CHARLS cohort, 413 participants (7.07 %) developed CMM during a median follow-up period of 6 years. Importantly, in both cohorts, the incidence of CMM progressively increased across increasing tertiles of BMI variability in the overall population (all *P* for trend <0.05). This trend was also observed within the baseline overweight and obese subgroups (all *P* for trend <0.05). Conversely, in subgroups with baseline BMI <25 kg/m^2^ (UK Biobank) or baseline BMI <24 kg/m^2^ (CHARLS), the incidence of CMM did not increase with increasing tertiles of BMI variability (Fig. 1, Table S2, Table S3).

### 3.3 Association of BMI variability with CMM risk in baseline overweight and obese population

In the UK Biobank, among participants who were overweight or obese at baseline (BMI ≥25 kg/m^2^), those in the highest tertile of BMI variability exhibited a higher burden of CMM compared to those in the lowest tertile in the fully adjusted model (model 2). The hazard ratios and 95 % confidence intervals were as follows: 2.97 (1.98–4.48) for SD, 3.06 (2.03–4.61) for CV, 2.90 (1.93–4.37) for VIM, and 2.45 (1.63–3.69) for ARV (Table 3). Similarly, in the CHARLS study, among individuals who were overweight or obese at baseline (BMI ≥24 kg/m^2^), higher BMI variability was associated with an increased hazard of CMM. Specifically, the HR for SD was 1.56 (95 % CI: 1.09, 2.35) after adjusting for all potential confounders in model 2, although this association was not observed for other measures of BMI variability, such as CV, VIM, and ARV (Table 4).

**Table 3:** Association of BMI variability with incident CMM in baseline BMI ≥25 kg/m^2^ subgroup (UK Biobank).

Tertiles of BMI variability	Crude Model		Model 1		Model 2	
	HR (95 % CI)	*P* value	HR (95 % CI)	*P* value	HR (95 % CI)	*P* value
**SD**						
**Lowest**	1(Reference)		1(Reference)		1(Reference)	
**Middle**	1.60 (0.95–2.70)	0.076	1.64 (1.11–2.43)	**0.014**	1.44 (0.93–2.24)	0.104
**Highest**	2.23 (1.36–3.64)	**0.001**	2.92 (2.02–4.24)	**<0.001**	2.97 (1.98–4.48)	**<0.001**
***P* for trend**		**<0.001**		**<0.001**		**<0.001**
**CV**						
**Lowest**	1(Reference)		1(Reference)		1(Reference)	
**Middle**	1.42 (0.85–2.37)	0.176	1.56 (1.05–2.32)	**0.028**	1.47 (0.95–2.29)	0.085
**Highest**	1.99 (1.23–3.21)	**0.005**	2.66 (1.83–3.86)	**<0.001**	3.06 (2.03–4.61)	**<0.001**
***P* for trend**		**0.004**		**<0.001**		**<0.001**
**VIM**						
**Lowest**	1(Reference)		1(Reference)		1(Reference)	
**Middle**	1.46 (0.89–2.41)	0.135	1.60 (1.08–2.36)	**0.019**	1.65 (1.07–2.55)	**0.023**
**Highest**	1.80 (1.12–2.90)	**0.016**	2.34 (1.61–3.41)	**<0.001**	2.90 (1.93–4.37)	**<0.001**
***P* for trend**		**0.016**		**<0.001**		**<0.001**
**ARV**						
**Lowest**	1(Reference)		1(Reference)		1(Reference)	
**Middle**	1.37 (0.83–2.25)	0.214	1.37 (0.92–2.03)	0.120	1.35 (0.87–2.08)	0.182
**Highest**	1.74 (1.09–2.79)	**0.021**	2.22 (1.53–3.23)	**<0.001**	2.45 (1.63–3.69)	**<0.001**
***P* for trend**		**0.020**		**<0.001**		**<0.001**

Abbreviation: HR, hazard ratio; CI, confidence interval; BMI,body mass index; SD,standard deviation; CV,coefficient of variation; VIM,variability independent of the mean; ARV,average real variability.

Crude Model: non-adjusted.

Model 1: adjusted for age, gender, education level, and marital status.

Model 2: adjusted for age, gender, education level, marital status, smoking status, drinking status, waist circumference, eGFR, TC, TG, HDL-C, LDL-C, SBP, DBP, lipid-lowering medications, antihypertensive medications, mean BMI, and baseline BMI.

**Table 4:** Association of BMI variability with incident CMM in baseline BMI ≥24 kg/m^2^ subgroup (CHARLS).

Tertiles of BMI variability	Crude Model		Model 1		Model 2	
	HR (95 % CI)	*P* value	HR (95 % CI)	*P* value	HR (95 % CI)	*P* value
**SD**						
**Lowest**	1(Reference)		1(Reference)		1(Reference)	
**Middle**	1.24 (0.88–1.76)	0.219	1.25 (0.88–1.77)	0.213	1.37 (0.92–2.04)	0.120
**Highest**	1.56 (1.12–2.17)	**0.008**	1.52 (1.09–2.12)	**0.013**	1.56 (1.09–2.35)	**0.017**
***P* for trend**		**0.007**		**0.011**		**0.017**
**CV**						
**Lowest**	1(Reference)		1(Reference)		1(Reference)	
**Middle**	1.16 (0.85–1.57)	0.360	1.15 (0.85–1.57)	0.364	1.24 (0.87–1.76)	0.235
**Highest**	1.22 (0.89–1.67)	0.229	1.19 (0.86–1.63)	0.294	1.26 (0.87–1.82)	0.221
***P* for trend**		0.227		0.292		0.216
**VIM**						
**Lowest**	1(Reference)		1(Reference)		1(Reference)	
**Middle**	1.15 (0.84–1.57)	0.382	1.15 (0.84–1.57)	0.383	1.23 (0.86–1.75)	0.259
**Highest**	1.27 (0.93–1.74)	0.133	1.24 (0.91–1.70)	0.177	1.31 (0.91–1.88)	0.150
***P* for trend**		0.133		0.178		0.151
**ARV**						
**Lowest**	1(Reference)		1(Reference)		1(Reference)	
**Middle**	0.80 (0.57–1.12)	0.195	0.80 (0.57–1.12)	0.195	0.92 (0.63–1.34)	0.648
**Highest**	1.12 (0.82–1.52)	0.474	1.09 (0.80–1.48)	0.589	1.12 (0.79–1.60)	0.524
***P* for trend**		0.344		0.445		0.456

Abbreviation: HR, hazard ratio; CI, confidence interval; BMI,body mass index; SD,standard deviation; CV,coefficient of variation; VIM,variability independent of the mean; ARV,average real variability.

Crude Model: non-adjusted.

Model 1: adjusted for age, gender, education level, and marital status.

Model 2: adjusted for age, gender, education level, marital status, smoking status, drinking status, waist circumference, eGFR, TC, TG, HDL-C, LDL-C, SBP, DBP, lipid-lowering medications, antihypertensive medications, mean BMI, and baseline BMI.

### 3.4 Association of BMI variability with CMM risk in baseline lean population

In contrast to the findings observed in the baseline overweight and obese population, higher BMI variability was not significantly associated with CMM risk among baseline lean participants (BMI <25 kg/m^2^in the UK Biobank and BMI <24 kg/m^2^in CHARLS). This lack of association was consistent across all measures of BMI variability (SD, CV, VIM, and ARV) in the fully adjusted model 2 (Table S4 for UK Biobank, Table S5 for CHARLS).

## 4 Discussion

Using data from two large prospective cohorts, we investigated the relationship between BMI variability and incident CMM in middle-age and older population. In both UK Biobank and CHARLS cohorts, the incidence of CMM progressively increased across increasing tertiles of BMI variability, particularly in overweight and obese individuals. Conversely, this trend was not observed in lean subgroups. Furthermore, baseline BMI significantly modified the association between BMI variability and CMM risk. Specifically, greater BMI variability was significantly associated with an increased risk of CMM among individuals classified as overweight or obese at baseline, while no such association was observed among those lean individuals at baseline.

Extensive research has explored the relationship between BMI and various diseases, including cardiovascular disease (CVD) [[23], [24], [25]]. However, these studies predominantly rely on BMI as a static measure, capturing only baseline body weight status. In fact, BMI in individuals is rarely constant and often fluctuates significantly due to lifestyle factors such as diet and physical activity. Recognizing this limitation, our study calculated BMI variability using BMI values from multiple follow-up time points to investigate its association with CMM. Our findings revealed that greater BMI variability was significantly associated with an increased risk of CMM among individuals classified as overweight or obese at baseline.

There has been a long - standing debate regarding the relationship between body weight fluctuation and the risks of T2DM and CVD. The majority of studies have established a positive correlation between weight variability and CVD. For instance, a systematic review and meta-analysis indicated that body weight variability was linked to an increased risk of CVD, regardless of ethnicity or diabetes status [18]. This finding was further supported by a cohort study of U.S. veterans, which showed that higher BMI variability was a significant risk marker for adverse cardiovascular events, including nonfatal myocardial infarction, acute ischemic stroke and cardiovascular death [17]. The relationship between body weight variability and CVD was also prominent in individuals with T2DM. Multiple studies have demonstrated a strong link between high body weight variability and the development of cardiovascular complications in individuals with T2DM, specifically increased risks of myocardial infarction, stroke, and all-cause mortality [[26], [27], [28]]. Additionally, a cohort study from Korea suggested that body weight variability was an independent risk factor for diabetes [29]. However, some studies have failed to firmly establish the association between weight fluctuation and adverse events such as diabetes and coronary heart disease [30,31]. These prior studies predominantly focused on the impact of BMI variability on single diseases. However, it is well-established that in middle-aged and older populations, conditions such as diabetes, coronary heart disease, and stroke frequently co-occur. Therefore, focusing solely on single diseases may fail to comprehensively capture the complex health risks associated with BMI variability in these populations. Notably, research exploring the relationship between long-term BMI variability and CMM remains limited. Our study investigates CMM as a more holistic and clinically relevant outcome measure to better reflect and assess the overall disease burden associated with BMI variability.

The mechanisms underlying the association between BMI variability and CMM remain incompletely understood. Several hypotheses have been proposed to explain this association, including metabolic stress, hormonal dysregulation, and immune reprogramming. Firstly, the “repeated overshoot” theory suggests that weight cycling induces fluctuations in blood pressure, heart rate, sympathetic nervous system activity, glomerular filtration rate, and circulating levels of glucose and lipids, thereby imposing an additional burden on the cardiovascular system [32]. Secondly, significant weight fluctuations disrupt hormonal homeostasis, characterized by reduced satiety hormones (e.g., leptin) and elevated hunger hormones (e.g., ghrelin), driving increased caloric intake [33]. Furthermore, some animal studies have shown that repeated cycles of weight loss and regain can accelerate cardiovascular disease by reprogramming the immune system [34]. Specifically, sustained high-fat diet exposure induced plaque formation and neutrophil infiltration in the artery wall, while macrophages mediated protective processes against atherosclerosis; switching to a low-fat diet reduced neutrophils and increased resident macrophages, but re-exposure to high-fat diet led to a surge of pro-inflammatory neutrophils and depletion of protective macrophages, accelerating atherosclerosis [35,36].These potential mechanisms provide a plausible explanation for our findings.

Our study revealed no significant association between BMI variability and CMM risk in lean individuals at baseline. A potential explanation for this observation lies in the differential adipose tissue distribution between lean and obese individuals. In lean individuals, adipose tissue is primarily localized subcutaneously, which may provide a buffering effect, mitigating the metabolic impact of weight fluctuations. In contrast, obese individuals are characterized by expanded adipose tissue compartments in both subcutaneous and visceral depots. This expansion could render visceral organs more susceptible to metabolic perturbations triggered by BMI variability [37]. Furthermore, emerging evidence indicates the presence of an “obesity memory” within adipose tissue of obese individuals and animal models, even following weight reduction. This memory, manifested through transcriptional dysregulation and aberrant regulation of metabolic pathways, compromises cellular function and the cellular response to metabolic challenges [38]. In contrast, this adipose tissue memory is not exhibited by lean individuals.

There are several advantages in this study. Firstly, we investigated the association between long-term BMI variability and the risk of CMM from a dynamic perspective, rather than using a single time-point BMI. Secondly, this study involved two prospective cohorts from different countries, providing large sample sizes and rigorous study designs. Our findings were consistent across both cohorts, indicating the generality of our results. Thirdly, our findings highlight the clinical importance of not only monitoring current BMI but also tracking BMI fluctuations, which has significant implications for CMM prevention. Additionally, this study provides valuable insights for future research. While our analysis focused on BMI variability, we were unable to explore specific BMI trajectories. Subsequent studies could further investigate which BMI trajectories pose the greatest risk for CMM development.

This study also has several limitations. Firstly, despite of the effort to adjust for a series of covariables, other residual confounding or unmeasured variables may remain, such as diet and genetic susceptibility. Secondly, selection bias may have been introduced by excluding participants lost to follow-up. Thirdly, our definition of CMM was based on both practical data availability (diabetes mellitus, coronary heart disease, and stroke) and a focus on binary disease states. Hypertension was not included because categorizing elevated blood pressure as a binary variable would necessarily underestimate the true effect of blood pressure on chronic disease [39], and PVD was not included due to incomplete data. Additionally, the number of participants with CMM at baseline was too small in both cohorts (UK Biobank: n = 71; CHARLS: n = 155) to investigate associations between BMI variability and cardiovascular mortality or all-cause mortality. Future analyses should be conducted in larger CMM populations.

## 5 Conclusions

The association between BMI variability and incident CMM was modified by baseline BMI among middle-aged and older adults. Specifically, greater BMI variability was significantly associated with an elevated risk of CMM in individuals with pre-existing overweight or obesity. Conversely, no significant association between BMI variability and CMM risk was observed in lean individuals at baseline. These findings highlight the clinical importance of minimizing BMI variability as a potential strategy for CMM risk reduction, particularly in individuals with pre-existing overweight or obesity.

## CRediT authorship contribution statement

**Xiaoying Ren:** Writing – original draft, Formal analysis, Data curation. **Mengge Yang:** Writing – original draft, Formal analysis, Data curation. **Juan Tian:** Methodology, Investigation. **Xiaona Chang:** Methodology, Investigation. **Guang Wang:** Writing – review & editing, Visualization. **Jia Liu:** Writing – review & editing, Visualization.

## Availability of data and materials

The CHARLS datasets used in this investigation are available in online repositories, and detailed descriptions of each survey and corresponding data are published at http://charls.pku.edu.cn/. The UK Biobank data are available from UK Biobank upon submission of a data request proposal (Application No. 425612).

## Ethics approval and consent to participate

The UK Biobank and CHARLS were approved by the North West Multi-Center Research Ethics Committee and Ethics Review Committees of Peking University, respectively. Each participant provided the informed consent in both cohorts.

## Consent for publication

Not applicable.

## Funding

This work was supported by grants from the Beijing Natural Science Foundation (No. L248018), the Chinese National Natural Science Foundation (No. 82072527), and the Beijing Hospitals Authority's Ascent Plan (No. DFL20220302).

## Declaration of competing interests

The authors declare no competing interests.
