# Supplementary material for: Long-term body mass index variability and the risk of cardiometabolic multimorbidity in middle-aged and older adults: Insights from two prospective cohorts
Source: Int J Cardiol Cardiovasc Risk Prev. 2025 Nov 17;27:200548. doi: 10.1016/j.ijcrp.2025.200548 (PMC12666677; doi:10.1016/j.ijcrp.2025.200548)
Supplement: Multimedia component 1 [file mmc1.docx]

**Appendix**

The formulas for calculating BMI variability metrics are as follows:

SD = $\sqrt{\frac{\sum_{i=1}^{n} {(BMI_{i}-meanBMI)}^{2}}{n}}$; The *meanBMI* represents the average of BMI values.

CV = $\frac{SD}{meanBMI}\times100\%$

VIM _=_ $\frac{SD}{{(meanBMI)}^{x}}$ ; Power x was modeled as “SD = k × mean*^x^*” and derived from fitting curves by nonlinear regression analysis implemented in the R studio.

ARV = $\frac{\sum_{i=1}^{n-1} |BMI_{i+1} - BMI_{i}|}{n-1}$


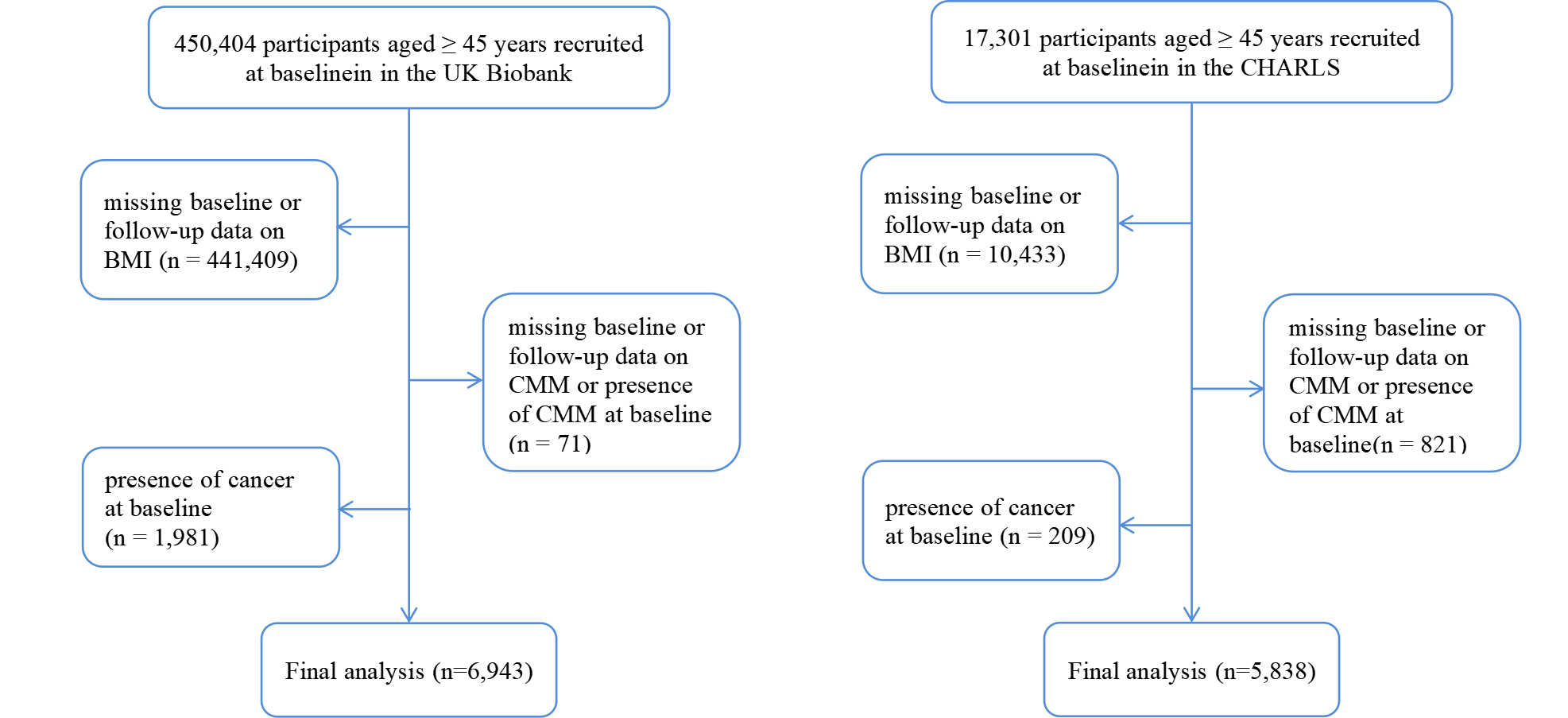


**Fig. S1.** Flowchart for the selection of study participants.

CHARLS: China Health and Retirement Longitudinal Study; CMM: cardiometabolic multimorbidity ; BMI: body mass index.

**Table S1.** The definitions of diabetes mellitus, coronary heart disease, and stroke in the UK Biobank.

| Disease | Data-fields | | |
| --- | --- | --- | --- |
|  | ICD-9 | ICD-10 | Self-reported |
| Diabetes mellitus | 41271 (250, 3572, 3620) | 41270 (E10-E14, G59.0, G63.2, H28.0,  H36.0, M14.2, N08.3), 130706, 130708, 130710, 130712, 130714 | 6153 (3), 6177 (3), 2443 (1), 2976, 20002 (1220,  1222, 1223) |
| Coronary heart disease | 41271(410-414) | 41270 (I20-I25, Z95.1, Z95.5), 131296,  131298, 131300, 131302, 131304,  131306 | 6150(1, 2), 3894, 3627, 20004(1070, 1095, 1523),  20002(1074, 1075) |
| Stroke | 41271 (3361, 3623, 430, 431, 4329, 4330, 4331, 4332, 4333, 4338, 4339, 434, 436) | 41270 (I60, I61, I62.9, I63, I64, I67.8,  I69.0, I69.3, G95.1, H34.1, H34.2,  S06.6), 131180, 131360, 131362,  131364, 131368, 131370, 131372,  131374, 131376, 131378 | 6150(3), 4056,  20002(1081, 1491, 1583, 1086) |

**Table S2.** The incidence of CMM in different tertiles of BMI variability in the UK Biobank cohort.

| **UK Biobank** | | **Tertiles of BMI variability** | | |
| --- | --- | --- | --- | --- |
|  |  | **Lowest** | **Middle** | **Highest** |
| **Total** | Total number | 2343 | 2296 | 2304 |
|  | The number of CMM | 62 | 30 | 44 |
|  | Perccent(%) | 2.65 | 1.31 | 1.91 |
|  | P for trend |  |  | 0.004 |
| **BMI ≥ 25kg/m^2^** | Total number | 1398 | 1398 | 1398 |
|  | The number of CMM | 26 | 39 | 47 |
|  | Perccent(%) | 1.86 | 2.79 | 3.36 |
|  | P for trend |  |  | 0.014 |
| **BMI < 25kg/m^2^** | Total number | 917 | 916 | 916 |
|  | The number of CMM | 3 | 12 | 9 |
|  | Perccent(%) | 0.33 | 1.31 | 0.98 |
|  | P for trend |  |  | 0.131 |

Abbreviation: BMI,body mass index; CMM,cardiometabolic multimorbidity.

**Table S3.** The incidence of CMM in different tertiles of BMI variability in the CHARLS cohort.

| **CHARLS** | | **Tertiles of BMI variability** | | |
| --- | --- | --- | --- | --- |
|  |  | **Lowest** | **Middle** | **Highest** |
| **Total** | Total number | 1948 | 1944 | 1946 |
|  | The number of CMM | 114 | 127 | 172 |
|  | Perccent(%) | 5.85 | 6.53 | 8.84 |
|  | P for trend |  |  | <0.001 |
| **BMI ≥ 24kg/m^2^** | Total number | 639 | 808 | 879 |
|  | The number of CMM | 52 | 81 | 109 |
|  | Perccent(%) | 8.14 | 10.02 | 12.40 |
|  | P for trend |  |  | 0.020 |
| **BMI < 24kg/m^2^** | Total number | 1252 | 1001 | 775 |
|  | The number of CMM | 58 | 42 | 45 |
|  | Perccent(%) | 4.63 | 4.20 | 5.81 |
|  | P for trend |  |  | 0.270 |

Abbreviation: BMI,body mass index; CMM,cardiometabolic multimorbidity.

**Table S4.** Association of BMI variability with incident CMM in baseline BMI < 25 subgroup (UK Biobank).

| **Tertiles of BMI variability** | **Crude Model** | | **Model 1** | | **Model 2** | |
| --- | --- | --- | --- | --- | --- | --- |
|  | **HR**  **(95% CI)** | ***P***  **value** | **HR**  **(95% CI)** | ***P***  **value** | **HR**  **(95% CI)** | ***P***  **value** |
| **SD** |  |  |  |  |  |  |
| **Lowest** | 1(Reference) |  | 1(Reference) |  | 1(Reference) |  |
| **Middle** | 1.01 (0.35-2.87) | 0.988 | 0.93 (0.38-2.23) | 0.865 | 0.71 (0.28-1.82) | 0.478 |
| **Highest** | 1.42 (0.54-3.72) | 0.480 | 1.75 (0.78-3.95) | 0.175 | 1.23 (0.52-2.90) | 0.637 |
| ***P* for trend** |  | 0.466 |  | 0.245 |  | 0.543 |
| **CV** |  |  |  |  |  |  |
| **Lowest** | 1(Reference) |  | 1(Reference) |  | 1(Reference) |  |
| **Middle** | 1.01 (0.35-2.87 ) | 0.990 | 1.12 (0.46 -2.70) | 0.807 | 0.83 (0.32-2.15) | 0.703 |
| **Highest** | 1.42 (0.54 -3.73) | 0.478 | 1.92 (0.85-4.33) | 0.114 | 1.35 (0.58-3.17) | 0.486 |
| ***P* for trend** |  | 0.464 |  | 0.181 |  | 0.575 |
| **VIM** |  |  |  |  |  |  |
| **Lowest** | 1(Reference) |  | 1(Reference) |  | 1(Reference) |  |
| **Middle** | 1.15 (0.42-3.17) | 0.787 | 1.36 (0.58-3.17) | 0.482 | 0.94 (0.38-2.31) | 0.895 |
| **Highest** | 1.27 (0.47-3.41) | 0.635 | 1.73 (0.76-3.96) | 0.194 | 1.26 (0.53-3.01) | 0.601 |
| ***P* for trend** |  | 0.635 |  | 0.275 |  | 0.674 |
| **ARV** |  |  |  |  |  |  |
| **Lowest** | 1(Reference) |  | 1(Reference) |  | 1(Reference) |  |
| **Middle** | 1.73 (0.63-4.75 ) | 0.290 | 1.69 (0.75-3.81) | 0.204 | 2.58 (1.11-6.01) | 0.028 |
| **Highest** | 1.33 (0.46-3.84 ) | 0.594 | 1.46 (0.63-3.40) | 0.386 | 1.13 (0.45-2.82) | 0.798 |
| ***P* for trend** |  | 0.620 |  | 0.487 |  | 0.725 |

Abbreviation: HR, hazard ratio; CI, confidence interval; BMI,body mass index; SD,standard deviation; CV,coefficient of variation; VIM,variability independent of the mean; ARV,average real variability.

Crude Model: non-adjusted.

Model 1: adjusted for age, gender,education level, and marital status.

Model 2: adjusted for age, gender, education level, marital status, smoking status, drinking status, waist circumference, eGFR, TC, TG, HDL-C, LDL-C, SBP, DBP, lipid-lowering medications, antihypertensive medications, mean BMI, and baseline BMI.

**Table S5.** Association of BMI variability with incident CMM in baseline BMI < 24 subgroup (CHARLS).

| **Tertiles of BMI variability** | **Crude Model** | | **Model 1** | | **Model 2** | |
| --- | --- | --- | --- | --- | --- | --- |
|  | **HR**  **(95% CI)** | ***P***  **value** | **HR**  **(95% CI)** | ***P***  **value** | **HR**  **(95% CI)** | ***P***  **value** |
| **SD** |  |  |  |  |  |  |
| **Lowest** | 1(Reference) |  | 1(Reference) |  | 1(Reference) |  |
| **Middle** | 0.90 (0.61-1.34) | 0.617 | 0.90 (0.61-1.34) | 0.609 | 0.86 (0.55-1.33) | 0.496 |
| **Highest** | 1.26 (0.85-1.86) | 0.243 | 1.17 (0.79-1.74) | 0.422 | 1.08 (0.69-1.68) | 0.749 |
| ***P* for trend** |  | 0.297 |  | 0.479 |  | 0.835 |
| **CV** |  |  |  |  |  |  |
| **Lowest** | 1(Reference) |  | 1(Reference) |  | 1(Reference) |  |
| **Middle** | 0.84 (0.56-1.26) | 0.404 | 0.84 (0.56-1.26) | 0.395 | 0.85 (0.54-1.33) | 0.471 |
| **Highest** | 1.09 (0.74-1.60) | 0.659 | 1.01 (0.69-1.48) | 0.964 | 1.00 (0.64-1.55) | 0.984 |
| ***P* for trend** |  | 0.678 |  | 0.975 |  | 0.952 |
| **VIM** |  |  |  |  |  |  |
| **Lowest** | 1(Reference) |  | 1(Reference) |  | 1(Reference) |  |
| **Middle** | 0.85 (0.57-1.28) | 0.442 | 0.86 (0.57-1.29) | 0.457 | 0.87 (0.55-1.36) | 0.535 |
| **Highest** | 1.14 (0.78-1.67) | 0.509 | 1.05 (0.72-1.55) | 0.788 | 1.03 (0.66-1.59) | 0.913 |
| ***P* for trend** |  | 0.532 |  | 0.805 |  | 0.946 |
| **ARV** |  |  |  |  |  |  |
| **Lowest** | 1(Reference) |  | 1(Reference) |  | 1(Reference) |  |
| **Middle** | 1.11 (0.76-1.62) | 0.605 | 1.10 (0.75-1.61) | 0.618 | 0.94 (0.62-1.43) | 0.770 |
| **Highest** | 1.10 (0.73-1.66) | 0.640 | 1.02 (0.68-1.54) | 0.921 | 0.97 (0.60-1.54) | 0.885 |
| ***P* for trend** |  | 0.610 |  | 0.873 |  | 0.855 |

Abbreviation: HR, hazard ratio; CI, confidence interval; BMI,body mass index; SD,standard deviation; CV,coefficient of variation; VIM,variability independent of the mean; ARV,average real variability.

Crude Model: non-adjusted.

Model 1: adjusted for age, gender,education level, and marital status.

Model 2: adjusted for age, gender, education level, marital status, smoking status, drinking status, waist circumference, eGFR, TC, TG, HDL-C, LDL-C, SBP, DBP, lipid-lowering medications, antihypertensive medications, mean BMI, and baseline BMI.
